# Tumor Extracellular Matrix Stiffness Promptly Modulates the Phenotype and Gene Expression of Infiltrating T Lymphocytes

**DOI:** 10.3390/ijms22115862

**Published:** 2021-05-30

**Authors:** Maila Chirivì, Fabio Maiullari, Marika Milan, Dario Presutti, Chiara Cordiglieri, Mariacristina Crosti, Maria Lucia Sarnicola, Andrea Soluri, Marina Volpi, Wojciech Święszkowski, Daniele Prati, Marta Rizzi, Marco Costantini, Dror Seliktar, Chiara Parisi, Claudia Bearzi, Roberto Rizzi

**Affiliations:** 1 Fondazione Istituto Nazionale di Genetica Molecolare, 20122 Milan, Italy; chirivi@ingm.org (M.C.); maiullari@ingm.org (F.M.); milan@ingm.org (M.M.); cordiglieri@ingm.org (C.C.); crostimariacristina@ingm.org (M.C.); sarnicola@ingm.org (M.L.S.); claudia.bearzi@cnr.it (C.B.); 2 Department of Medical Surgical Sciences and Biotechnologies, Sapienza University of Rome, C.so della Repubblica 79, 04100 Latina, Italy; 3 Department of Biology, University of Rome Tor Vergata, 00133 Rome, Italy; 4 Institute of Biochemistry and Cell Biology, National Research Council of Italy (IBBC-CNR), Via Ercole Ramarini, 32, Monterotondo, 00015 Rome, Italy; asoluri10@gmail.com (A.S.); chiara.parisi82@gmail.com (C.P.); 5 Institute of Physical Chemistry Polish Academy of Sciences, Marcina Kasprzaka 44/52, 01-224 Warszawa, Poland; presuttidario@gmail.com (D.P.); mcostantini@ichf.edu.pl (M.C.); 6 Unit of Molecular Neurosciences, University Campus Bio-Medico, 00128 Roma, Italy; 7 Faculty of Materials Science and Engineering, Warsaw University of Technology, 02-507 Warsaw, Poland; marina.volpi@pw.edu.pl (M.V.); wojciech.swieszkowski@pw.edu.pl (W.Ś.); 8 Department of Transfusion Medicine and Hematology, IRCCS Granda Hospital Maggiore Policlinico Foundation, Via Francesco Sforza 35, 20122 Milan, Italy; daniele.prati@policlinico.mi.it; 9 Ufficio Programmazione e Grant Office, National Research Council of Italy (UPGO-CNR), Piazzale Aldo Moro 7, 00185 Rome, Italy; marta.rizzi@cnr.it; 10 Department of Biomedical Engineering, Technion Institute, Haifa 32000, Israel; dror@bm.technion.ac.il; 11 Institute of Genetic and Biomedical Research, UOS of Milan, National Research Council (IRGB-CNR), Via Gaudenzio Fantoli 16/15, 20138 Milan, Italy; 12 Institute of Biomedical Technologies, National Research Council (ITB-CNR), Via Fratelli Cervi, 93, Segrate, 20090 Milan, Italy

**Keywords:** tumor microenvironment, extracellular matrix, T lymphocytes, 3D culture

## Abstract

The immune system is a fine modulator of the tumor biology supporting or inhibiting its progression, growth, invasion and conveys the pharmacological treatment effect. Tumors, on their side, have developed escaping mechanisms from the immune system action ranging from the direct secretion of biochemical signals to an indirect reaction, in which the cellular actors of the tumor microenvironment (TME) collaborate to mechanically condition the extracellular matrix (ECM) making it inhospitable to immune cells. TME is composed of several cell lines besides cancer cells, including tumor-associated macrophages, cancer-associated fibroblasts, CD4^+^ and CD8^+^ lymphocytes, and innate immunity cells. These populations interface with each other to prepare a conservative response, capable of evading the defense mechanisms implemented by the host’s immune system. The presence or absence, in particular, of cytotoxic CD8^+^ cells in the vicinity of the main tumor mass, is able to predict, respectively, the success or failure of drug therapy. Among various mechanisms of immunescaping, in this study, we characterized the modulation of the phenotypic profile of CD4^+^ and CD8^+^ cells in resting and activated states, in response to the mechanical pressure exerted by a three-dimensional *in vitro* system, able to recapitulate the rheological and stiffness properties of the tumor ECM.

## 1. Introduction

A key feature of the immune system is the continuous motility and migratory behavior of lymphocytes, as a patrol of the body districts, until the identification of the appropriate microenvironments for maturation, differentiation and survival [1]. This process of targeted circulation and localization is called homing, and it is crucial for orchestrating immune responses. As long as the lymphocytes are not activated by foreign antigens, they continuously recirculate between the blood and lymphoid organs; a process called lymphocyte trafficking [2]. These cells that have not yet encountered the antigen are termed naïve (N) cells [3]. Recirculation ends when the first meeting between a T cell and its specific antigen occurs in the secondary lymphoid organs [4]. This event, called priming, requires the interaction of the T cell with an antigen-presenting cell (APC) [5] that has internalized and processed the antigen itself and exposes the peptides on its surface bound to the autologous and highly polymorphic glycoprotein molecules of the major histocompatibility complex (MHC) [6]. When the T cell receptor (TCR) recognizes its specific MHC peptide complex on the surface of an APC cell, a cascade of intracytoplasmic molecular events, that transduce the signal to the nucleus, is triggered allowing the activation, proliferation and differentiation of N cells into effector memory (EM) and central memory (CM) cells [7]. Once activated, lymphocytes become extremely heterogeneous from a functional and phenotypic point of view and capable of extremely complex biological responses and functions. Once they complete their differentiation into EM cells, they leave the secondary lymphoid organs through the efferent lymphatic vessels, which are recalled by the inflamed tissues [8]. EM cells compared to N cells express different adhesion molecules that no longer mediate the recirculation, but the migration of lymphocytes in the tissues of the infection, where the T lymphocytes recognize the antigen that caused their activation. Once completed their function, EM cells undergo apoptosis, with the exception of a small group that differentiates into CM cells, which remain dormant, even for long periods, until they receive a new specific antigenic stimulus [9]. During priming, CD4^+^ and CD8^+^ T cells can be polarized to distinct subpopulations of effector cells, such as EM CD8^+^ T cells, also commonly known as cytotoxic T lymphocytes [10] capable of counteracting or terminating tumor progression. On the other side, the tumor mass consists of heterogeneous populations of malignant and non-malignant cells incorporated in a pathological extracellular matrix (ECM) [11]. Cancer cells significantly alter the tumor microenvironment (TME) by activating tumor-associated macrophages (TAM) and cancer-associated fibroblasts (CAF), unbalancing the ratio between growth factors and cytokines, released in the ECM [12]. This awareness has led researchers to reconsider the best way to attack a solid tumor, and the intervention on stromal elements is becoming increasingly attractive [13]. To date, the extreme plasticity of TME is considered one of the emerging mechanisms by which the tumor attempts to evade the immune system or the pharmacological approaches [14]. The pharmacological resistances could be mediated by an immunosuppressive TME (iTME) [15]. Specifically, the alteration of mechanical properties of the ECM are important modulators of cell behavior and are correlated with biochemical cues, released from the TME, to regulate tumor progression, metastatic dissemination and immune evasion [16]. Recently, it has been shown that in triple-negative breast cancer (TNBC) both the molecular and mechanical components (stiffness) of the tumor-associated ECM (tECM) are capable to inhibit the tumor mass permeabilization by cytotoxic CD8^+^ T cells, whose presence close to cancer is generally associated with good prognosis and successful pharmacological therapy [17]. The degradation of the ECM-surrounding tumor and the subsequent deposition of a higher density and stiffness ECM [18], is an essential part of its invasiveness and progression, and it is one of the mechanisms responsible for the aggressiveness of the tumor [19]. In the last decade, numerous discoveries have highlighted how the physical properties of the ECM can influence the response to therapy, modulating both the tumor physiology [20] and the phenotype of T lymphocytes attempting to cope with cancer. Recently, a direct correlation has been found between tumor ECM density and poor prognosis in different pathological scenarios [21], also supported by further studies showing that an ECM with high rigidity can also induce malignant transformations of epithelial cells [22]. T lymphocytes respond to the mechanical properties of the surrounding ECM [23] by modulating their TCR-mediated activation [24]. In this study, we recapitulated the mechanical properties of the tumor ECM in three-dimensional culture assays to investigate whether the mechanical properties of the ECM can rapidly and directly influence the survival, phenotype and gene expression of lymphocytes. Poly(ethylene glycol)-fibrinogen (PEG-FB)-based hydrogels were used to simulate the stiffness of healthy breast tissue and the five times stiffer breast tumor matrix [25]. The stiffness was increased modulating the concentration of PEG-FB and adding 1% PEG diacrylate (PEG-DA) in the most rigid condition. PEG-FB is a biocompatible biomaterial widely used for cell migration studies [26,27] and three-dimensional cultures of healthy and cancer tissues [28,29].

## 2. Results

CD4^+^ and CD8^+^ T lymphocytes were isolated from fresh buffy coat derived from 3 healthy donors and were included in ad hoc generated three-dimensional (3D) matrices recapitulating, in vitro, both the physical and stiffness characteristics of the ECM [30] of healthy tissue (hECM) and triple-negative breast cancer (tECM): specifically, two different precursors PEG-FB based solutions were used [31]. At the end of the established experimental time point periods (0 h, 12 h and 24 h), the samples were analyzed.

**Figure 1:**
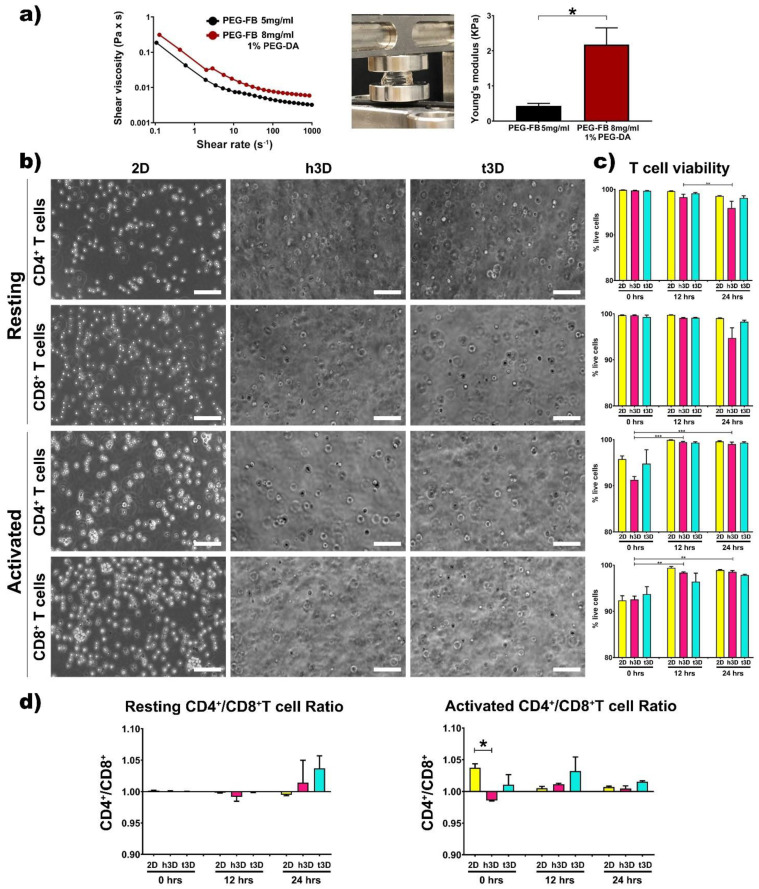
Hydrogel analysis and cell viability. (**a**) Rheological characterization of 5 mg/mL PEG-FB (black) and 8mg/mL PEG-FB supplemented with 1% PEG-DA (red) bio-assembled structures; compression tests and Young’s modulus evaluation of 3D bulks. (**b**) Representative bright field images of CD4^+^ and CD8^+^ T cells in standard culture condition (2D), in h3D and t3D constructs. Scale bars represent 100 μm. (**c**) Cell viability assessment after 0, 12 and 24 h of culture in different experimental conditions performed by FACS analysis. (**d**) CD4^+^/CD8^+^ T cell ratio in resting and activated state at 0, 12, 24 h. *n* = 3 for each experimental group. * *p* < 0.033, ** *p* < 0.002, *** *p* < 0.001.

The rheological properties of the two matrices were examined by a rotational rheometer. As expected, the precursor solution containing 8 mg/mL PEG-FB plus 1% PEG-DA appears to have a higher shear viscosity compared to the healthy one (Figure 1a).

Subsequently, the two precursor solutions were used to generate the healthy and cancerous constructs. The bulks generated after polymerization, obtained adding Irgacure 2959 and exposing the solutions for 5 min to UV light at low penetrance to avoid DNA damage, were subjected to mechanical testing to determine their elasticity in terms of kilopascals (Young’s modulus) [31]. As shown in Figure 1a, the stiffness of the two types of constructs resulted in 400 Pa for the healthy constructs (PEG-FB 5 mg/mL) and 2500 Pa for the cancer constructs (PEG-FB 8 mg/mL plus 1% PEG-DA). Afterward, the two types of hydrogels, with their specific mechanical characteristics, were used to encapsulate resting and activated CD4^+^ and CD8^+^ T lymphocytes (Figure 1b). First, we evaluated the viability of the T cells included in h3D and t3D bulks. The transition from the 2D to the 3D cultures did not affect cell viability within the 24 h in all the experimental conditions (Figure 1c). However, the CD4^+^/CD8^+^ viability ratio shows a trend in favor of CD4^+^ as shown in Figure 1d, with the exception of cells in resting condition at 12 h and in 2D group at 24 h. The difference in cell loss between CD4^+^ and CD8^+^ is statistically significant (*p* = 0.018) when cells are included in h3D versus those in 2D. The hypothesis is that the tumor mass holds CD4^+^ T cells close to itself to support a high level of inflammation while keeping CD8^+^ cytotoxic cells away, preserving tumor progression [32].

**Figure 2:**
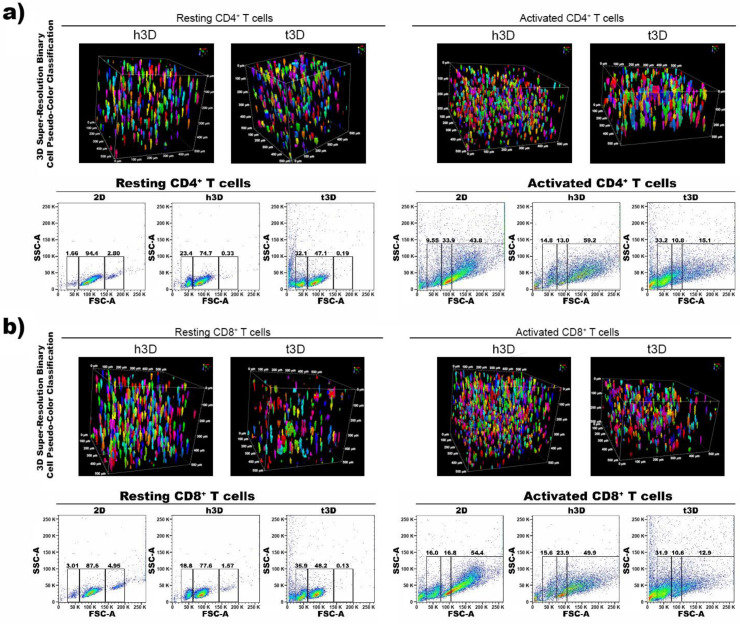
Qualitative and morphological analysis. (**a**) CD4^+^ and (**b**) CD8^+^ T cell size analysis. Pseudo-color object classification of T cells encapsulated in h3D and t3D hydrogels. Gates illustrate the percentage of cells with different sizes. The physical parameters were set using SSC-A and FSC-A. *n* = 3 for each experimental group.

Resting and activated CD4^+^ and CD8^+^ cells in h3D and t3D bulks were stained for Phalloidin while nuclei counterstained with Dapi to determine morphologic and nuclear changes in terms of dimensions. The T cells showed a similar orientation and distributions inside the two matrices even if the activated cells were prone to form clusters within hydrogels (Figure 2a,b).

Then, we evaluated the cell dimensions through FACS analysis (Side Scatter SSC-A and Forward Scatter FSC-A) at 0 h. Both the CD4^+^ and CD8^+^ populations in the resting phase inserted in the 3D matrices underwent an instant reduction in size, more marked when the surrounding matrix was stiffer. In particular, the percentage of most cells in h3D bulks displaying dimensions of 60,000–150,000 FSC-A was reduced compared to 2D condition (CD4^+^ = 74.7% vs. 94.4%; CD8^+^ = 77.6% vs. 87.6%), while the remaining cells had dimensions of 30,000–60,000 FSC-A. Conversely, lymphocytes in t3D presenting 60,000–150,000 FSC-A were reduced compare to 2D culture (CD4^+^ = 47.1% vs. 94.4%; CD8^+^ = 48.2% vs. 87.6%), while smaller-sized cells (30,000–60,000 FSC-A) were increased compared to 2D (CD4^+^ = 32.1% vs. 1.66%; CD8^+^ = 35.9% vs. 3.01%) (Figure 2a,b).

However, a different response occurred when CD4^+^ and CD8^+^ cells were in activated state. The inclusion of CD4^+^ lymphocytes in the matrices after their activation, induced an increase in cell volume from 100,000 to 250,000 FSC-A (43.8% vs. 59.2%), mostly when the cells were encapsulated in the hECM, which correlated with the activation procedure effect. Conversely, when CD4^+^ T cells were cultured in the tECM, they drastically reduced their cell size from 100,000 to 50,000 FSC-A (33% vs. 9.55%) (Figure 2a). Activated CD8^+^ T cells reduced also their size in h3D displaying 60,000–100,000 FSC-A (23.9% vs. 16.8%) and in t3D 30,000-60,000 FSC-A (31.9% vs. 16%) (Figure 2b).

**Figure 3:**
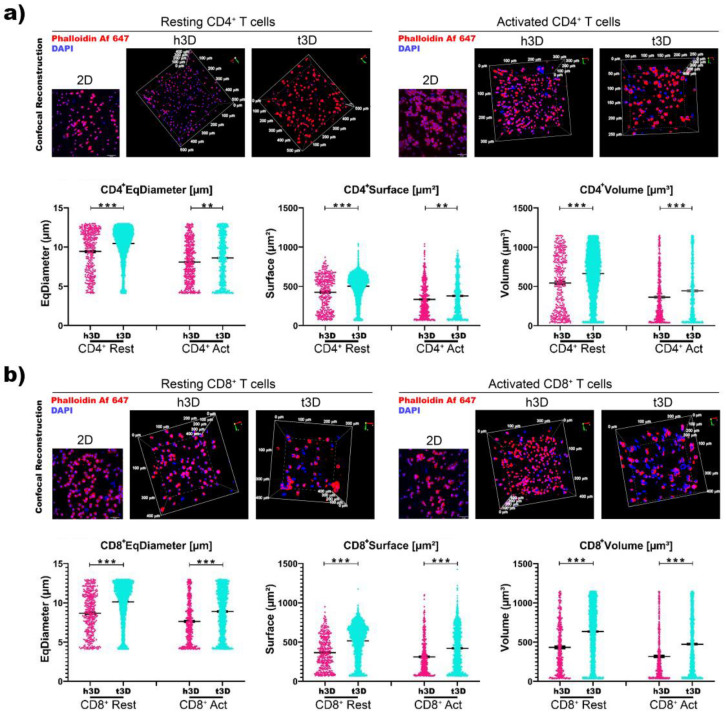
Volumetric analysis. (**a**) CD4^+^ and (**b**) CD8^+^ T cell nuclear dimension assay. Representative images of 2D samples and volumetric 3D reconstruction of h3D and t3D bulks stained for Phalloidin Alexa Fluor647. Nuclei were counterstained with Dapi. Scale bars represent 50 μm in 2D images. Scatter dot plot indicating T cell nucleus diameter (μm), surface area (μm^2^) and volume (μm^3^). Error bars represent ±SEM. Student’s t-test, ** *p* < 0.002, *** *p* < 0.001. *n* = 3 for each experimental group.

Surprisingly, when we correlated cell volume with its nuclear volume, we found an inverse response (Figure 3).

Volumetric 3D reconstruction was performed for in-depth visualization on 500 μm^3^ volumes and digital analysis was used to evaluate the nuclei diameter (μm), surface (μm^2^) and volume (μm^3^) of each cell in all conditions (Figure 3). CD4^+^ and CD8^+^ T cells in both resting and activated state displayed a significative increase in the three nuclei parameter when included in t3D compare to the h3D (Figure 3a,b).

**Figure 4:**
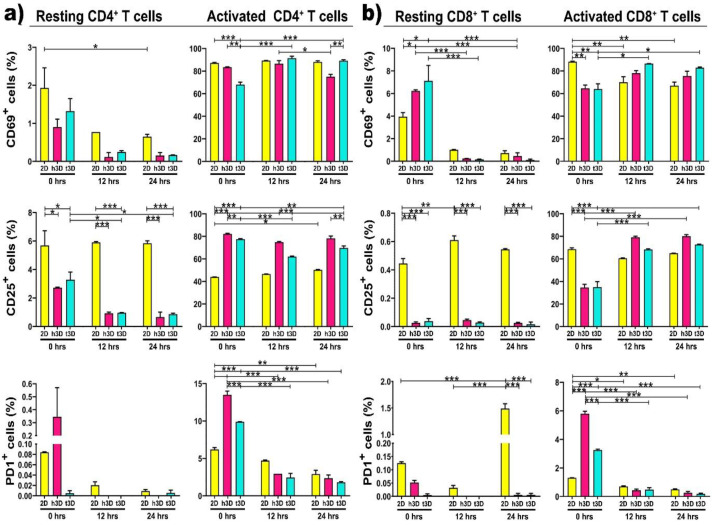
Surface markers expression. Graphs represent the percentage of resting and activated CD4+ (**a**,**b**) CD8+ T cells expressing CD69 (upper panel), CD25 (middle panel) and PD1 (lower panel) surface markers. * *p* < 0.033, ** *p* < 0.002, *** *p* < 0.001 were considered statistically significant. *n* = 3 for each experimental group.

Next, we analyzed the expression of the early CD69 and the late CD25 activation markers based on surrounding environment increased mechanical pressure, in both resting and activated CD4^+^ and CD8^+^ T cells. As expected, resting CD4^+^ cells, cultured in 2D, expressed CD69 at a very low level (2%), but the encapsulation into 3D matrices completely silences the expression of this activation marker along 24 h. The same tendency was also detected for the CD25 expression, which unlike CD69, remains expressed at 6% in 2D cultures for all 24 h. PD-1 expression, as predictable, was not detected in any of the culture conditions, either in 2D or h3D and t3D (Figure 4a, left panels). What emerges from the initial experiment performed in different environments is that the hardness of the matrix or the culture condition does not immediately change the antigenic profile of resting CD4^+^ cells.

We have not noticed an equal trend between the different conditions and the three different surface markers, which makes us assume that their expression did not always changes instantaneously. Specifically, we observed that the expression of CD69 did not change in CD4^+^ T cells in both resting and activated state. The surface marker CD25 decreased in resting cells and increases in activated ones, while PD1 expression tended to increase at 0 h, although not significantly. Instead, the percentage of CD8^+^ T cells positive for CD69 increased in the resting state but decreased in the activated condition. Finally, CD25 was reduced both in resting and in activated conditions, while the expression of PD1 was increased only in the activated state of CD8^+^ T cells.

The first significant result was that the activated CD4^+^ cells, inserted in t3D constructs, significantly increased the expression of CD69 compared to those embedded in the healthy matrix after 24 h of culture (*p* = 0.003), while CD25 expression was upregulated in the h3D compared to the tumor one, starting from the moment in which the lymphocytes were inserted in the 3D culture (0 h *p* = 0.010; 12 h *p* < 0.001; 24 h *p* < 0.003) (Figure 4a, right panels). This outcome allowed us to speculate that the stiffness of the matrix induces CD4^+^ T cells slow deactivation. A further analysis was conducted on the expression of PD-1. PD-1 is a member of the immunoglobulin family and its ligation appears to provide inhibitory signals that dampen T-cell receptor (TCR) signaling [33]. An instantaneous expression of PD-1 was detected in the inserted CD4^+^ cells in the 3D arrays compared to the 2D culture. However, its expression was immediately downregulated in all culture conditions at both 12 and 24 h (*p* < 0.001) (Figure 4a, right panels).

Subsequently, we also evaluated resting CD8^+^ T lymphocytes, which were shown to lose the ability to reach tumor mass in microenvironments conditioned by certain tumors, such as triple negative breast cancer [34]. Non-activated CD8^+^ lymphocytes behave similar to resting CD4^+^ cells: indeed, they did not express CD69, CD25 and PD-1 markers after the extraction from peripheral blood and the sorting procedure (Figure 4b, left panels). This data reveals that T lymphocytes are more plastic to the mechanical conditioning of the ECM, when they are already in the activation state compared to the resting condition.

Regarding activated CD8^+^ lymphocytes, we observed that CD69 expression was upregulated in lymphocytes inserted in the harder matrix than in all other conditions. The expression of the CD25 marker displayed a tendency to increase in cells that have been included in the matrix, compared to cells cultured in the 2D system. CD8^+^ in h3D and t3D exhibited an increase of the late activation marker over time, but, surprisingly, when included in a stiffer matrix, than in the healthy one, they showed a loss of activation (Figure 4b, right panels). These results suggest that CD25 expressing T cells represent an early stage in the differentiation of CD8^+^ cells. Accumulation of this cell phenotype in older people is a prerequisite for an intact immune response in the absence of N T cells [35]. The presence of PD-1 traced the same path in CD4^+^ cells: after an initial hyperactivation in the 3D matrices, it was downregulated at 24 h in all culture conditions (*p* < 0.001) (Figure 4b, right panel).

**Figure 5:**
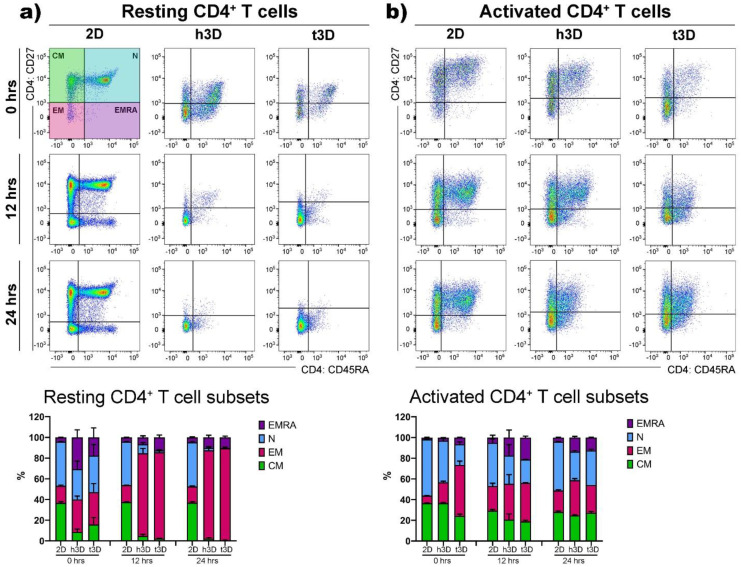
CD4^+^ T cell subsets. (**a**) Resting and (**b**) activated CD4^+^ T cells were gated on CD27 and CD45RA markers to determine the CM, N, EM and EMRA subsets as shown in the quadrant plots and related histograms under the different conditions at 0, 12 and 24 h. *n* = 3 for each experimental group.

Then, we detected the phenotypic features, i.e., N, EM, CM and EMRA cells, on the basis of their relative surface expression of CD27 and CD45RA molecules, in order to evaluate the stiffness impact on the T cell subsets. The result that emerged, embedding CD4^+^ cells in the 3D healthy and tumor matrices, was that the phenotypic heterogeneity found and maintained in the 2D cultures (Figure 5a, left panels) was lost in both experimental conditions (Figure 5a, right panels).

Besides, the resting lymphocytes significantly differentiated in the EM populations in both mechanical rigidities investigated. On the other hand, CD4^+^ cells activated for 48 h did not present the EMRA population that emerges after 24 h in the 2D culture condition in a percentage of about 10% (Figure 5b, left panels). Consequently, the insertion of activated CD4^+^ cells into 3D arrays instantly increased the expression of the effector memory (EM) subtype (h3D = 20%; t3D = 50%) (Figure 5b, right panels). Furthermore, the cells included in the t3D bulks upregulated the expression of EM after 12 h of culture compared to the 2D condition (2D = 7%; t3D = 37%), almost silencing the expression of the EMRA phenotype, which appeared to the extent of 10% after 24 h in the 2D system. After 24 h of culture the phenotype selection clearly showed an increase of 10% and 6% of EM cells in h3D and t3D matrices respectively, compared to the 2D condition (Figure 5b, right panels).

**Figure 6:**
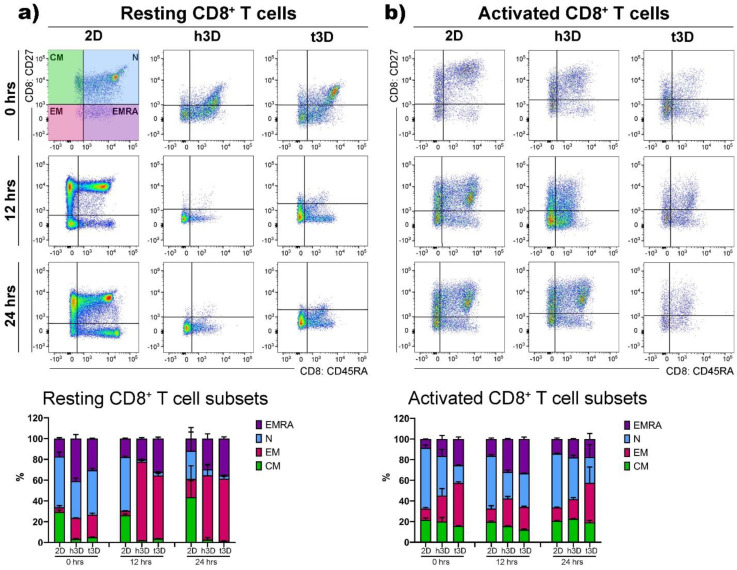
**CD8^+^ T cell subsets.** (**a**) Resting and (**b**) activated CD8^+^ T cells were gated on CD27 and CD45RA markers to evaluate the CM, N, EM and EMRA subsets as indicated in the boxplots and related histograms in the different conditions at 0, 12 and 24 h of culture. *n* = 3 for each experimental group.

Resting CD8^+^ cells behaved similar to their CD4^+^ counterpart significantly upregulating EM differentiation after their introduction into 3D matrices (2D = 3%; h3D = 20%; t3D = 21%), while maintaining a marked presence of the terminal differentiated EMRA phenotype, compared to the 2D condition (2D = 17%; h3D = 41%; t3D =30%) (Figure 6a).

Activated CD8^+^ cells, inserted in 3D matrices, especially in the more rigid t3D, instantly displayed the EM phenotype to the detriment of the EMRA phenotype. After 24 h, the situation was similar in the two matrices, while the cells grown in the 2D system maintained an increase of 20% in N phenotype respect to the 3D cultures (30% vs. 50%) (Figure 6b).

**Figure 7:**
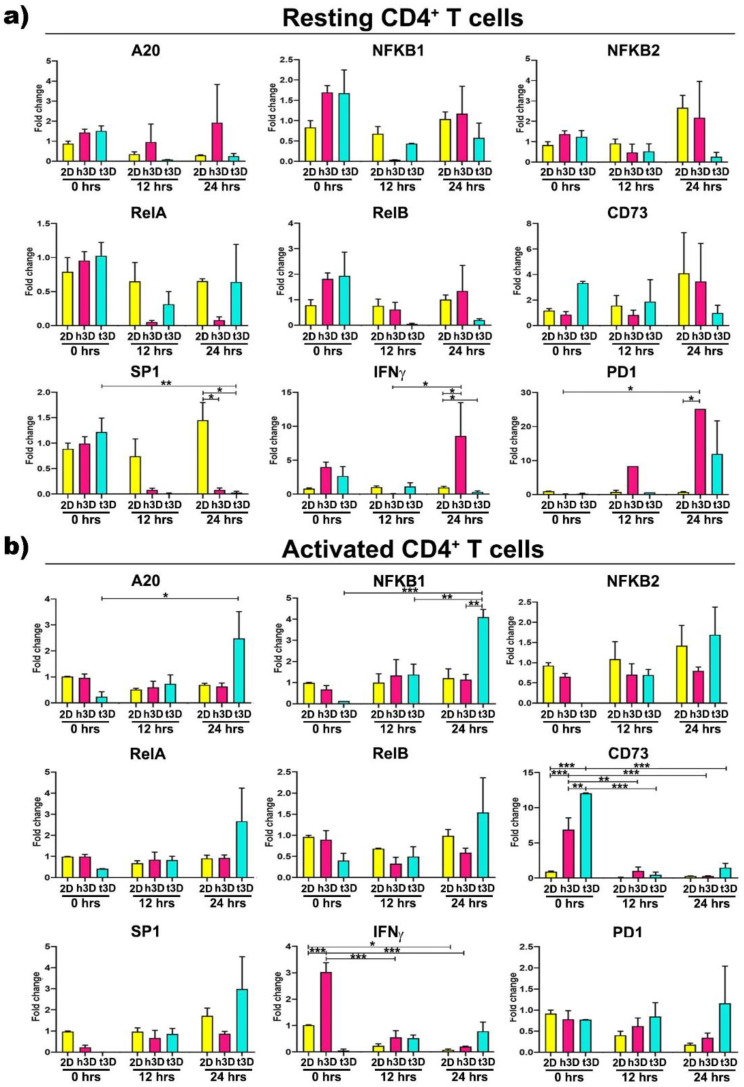
Gene expression assessment of CD4^+^ T cells. Histograms related to the canonical NF-kB pathway, inflammation and exhaustion genes in CD4^+^ T cells (**a**) in resting and (**b**) in activated state. Error bars represent ±SEM. Repeated measure ANOVA with Tukey correction was used to evaluate the differences between means; * *p* < 0.033, ** *p* < 0.002, *** *p* <0.001. *n* = 3 for each experimental group.

Then, in order to explore the sudden molecular changes induced by the different mechanical properties of the 3D environment, with respect to the 2D culture condition, we analyzed lymphocytes for gene expression of molecules known to be involved in tumor immune response in the different culture conditions. The major effects in resting CD4^+^ cells were observed after 24 h of 3D culture (Figure 7a).

At this time point, the transcription factor *SP1* was downregulated in both the h3D and t3D compared to 2D culture (h3D vs. 2D *p* = 0.022; t3D *p* = 0.007), while *IFNγ* appeared to follow the opposite trend, being upregulated in the healthy (h3D vs. 2D *p* = 0.021) and downregulated in the stiffer condition (t3D vs. 2D *p* = 0.023). A marked increase of *PD1* gene was also observed in CD4^+^ cells after 24 h in the healthy 3D matrix (h3D vs. 2D *p* = 0. 012) (Figure 7a).

Afterward, we characterized the abrupt change in gene expression, determined by the mechanical properties of the surrounding ECM, in activated CD4^+^ cells. In these cells, the stiffer matrix significantly acted on the expression of the NF-kB canonical pathway genes. In particular, *A20* and *NFKB1* increased over time, reaching the maximum after 24 h of culture (*NFKB1* in t3D vs. 2D *p* = 0.002), together with a marked tendency to *RelA* overexpression at the same time (Figure 7b). Furthermore, activated CD4^+^ lymphocytes instantaneously upregulated the *CD73* marker (*p* < 0.001), when inserted in both 3D matrices, with enhanced gene expression maintained only in the most rigid ECM condition, along the 24 h. The healthy matrix, on the other hand, is able to downregulate the expression of *IFNγ* in activated CD4^+^ lymphocytes, after an instantaneous boosting when the cells were inserted into the hydrogel.

**Figure 8:**
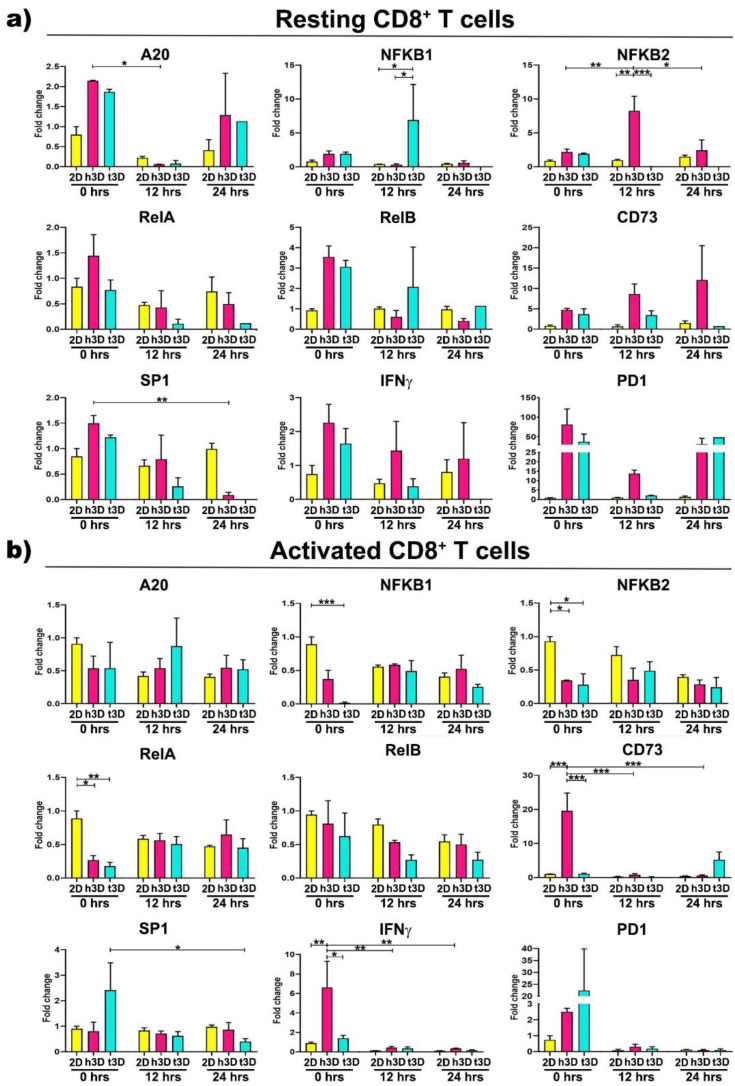
Gene expression assessment of CD8^+^ T cells. CD8^+^ T cells in (**a**) resting and (**b**) activated condition evaluated by qRT-PCR. The canonical NF-kB pathway, inflammation and exhaustion genes were analysed. Error bars represent ±SEM. Repeated-Measure ANOVA with Tukey correction was used to evaluate the differences between means; * *p* < 0.033, ** *p* < 0.002, *** *p* < 0.001. *n* = 3 for each experimental group.

Expression changes in *NF-kB* genes also characterized CD8^+^ lymphocytes. Resting cells exposed to healthy matrix showed *A20* gene downregulation concomitant with *NFKB2* growing expression after 12 h of culture (h3D vs. 2D *p* = 0.001), while in the tumor condition at the same time point it was observed an *NFKB1* increase (t3D vs. h3D *p* = 0.043) in contrast to a marked *NFKB2* decrease (t3D vs. h3D *p* < 0.001). *SP1* expression was instead downregulated over time in the h3D condition, reaching significance after 24 h in culture (*p* = 0.004) (Figure 8a).

On the other hand, most changes in gene expression observed in activated CD8^+^ population occurred rapidly, principally at 0 h in 3D matrices, particularly in the stiffer one. At this time point, *NFKB2* and *RelA* genes were strongly reduced in both healthy and tumor conditions (*NFKB2* in h3D vs. 2D *p* = 0.023; t3D vs. 2D *p* = 0.014; *RelA* in h3D vs. 2D *p* = 0.020; t3D vs. 2D *p* = 0.006), while the t3D samples also downregulated *NFKB1* (*p* < 0.001). The t3D system also reduces the expression of *SP1* at 24 h (*p* = 0.044), after an initial trend of upregulation (Figure 8b). Concomitant regulation of both *NF-kB* and *SP1* transcription factors is known to occur in particular areas of the gene promoters responsible for remodeling the ECM.

Together with the previous transcription factors, 3D matrices immediately affect also *CD73* and *IFNγ* gene expression with an opposite effect between healthy and tumor conditions. In fact, the upregulation of *CD73* and *IFNγ* observed in the healthy matrix opposes to a significant reduction of both genes in the tumor condition. These reductions make us to speculate that tECM is able to promote the deactivation of cytotoxic CD8^+^ cells as they approach the tumor mass.

## 3. Discussion

Activation of the immune system is the first mechanism exerted by the organism to tackle the onset of cancer, and to date it is considered the most powerful weapon available to limit neoplastic progression. Priming process promotes the activation of naïve T cells, cells with an enlarged nucleus containing diffuse chromatin, prominent nucleoli, abundant mitochondria-rich cytoplasm and rough endoplasmic reticulum, all markers of cellular activity. Usually, the activation of T lymphocytes, responsible for adaptive immunity, requires the presentation by the cells of specific antigens classified within the innate immunity; the phenotype of these cells, depending on their previous history and the microenvironment, influences the lymphocyte activation process to the extent that it decrees the outcome. On the other hand, many mechanisms are activated by the tumors to cope with the immune action, ranging from the secretion of factors inhibiting the invasion of defined immune cells, such as cytotoxic CD8^+^, to a massive mechanical remodeling of the peri-tumoral ECM, that inhibits the infiltration of lymphocytes into the tumor ECM. In fact, the presence of cytotoxic CD8^+^ lymphocytes in the TME, and in the proximity of the tumor mass, anticipates not only a good prognosis, but is also able to convey the effects of pharmacological treatments towards success [10]. The lymphocytes migratory mechanism involves the infiltration between the pores of the ECM [20], as opposed to stem cells which invade the tissues by digesting the ECM through the secretion of metalloproteases 2 and 9 [36,37]. The reduction of the ECM pores, through the remodeling exerted by CAFs [18] could constitute an insurmountable obstacle for the lymphocytes trying to reach the tumor mass, since they cannot reduce the size of the nucleus below the pore size.

Among the immune escaping mechanisms performed by tumors, in this study we characterized the ability of the mechanical properties, exerted by tumoral matrices, to influence the viability, phenotype and gene expression of CD4^+^ and CD8^+^ lymphocytes, in resting and activated state. In particular, our principal goal was to characterize the changes in the immune cells profile in matrices of different stiffness. We noticed a sudden cell size reduction upon encapsulation of T cells into 3D matrices, triggered by 360-degree pressure on the cell membrane. The reduction in cell volume was significantly more marked when both lymphocyte populations were included in the tECM than in the healthy one. However, at the same time, there was an increase in the size of the nucleus in t3D compared to h3D samples, and surprisingly, the cells in resting state showed larger nuclear dimensions than the activated T cells. Recent studies have reported a negative correlation between the deformability of T cells, particularly CD4^+^ lymphocytes, and the ratio of nucleus to cell size.

Since deformability is a key feature related to T cell functions during extravasation and migration to surrounding tissues, our data suggest that the tECM-induced increase in CD8^+^ nucleus size represents the first line of mechanisms for the immune escape [38].

An important observation in this study was that T cells clearly respond to the density of the surrounding matrix. In particular, we analyzed the short culture times, and we found many differences in the 2D versus 3D conditions. For the phenotypic changes, characteristic of the differentiation of T lymphocytes, it is necessary to wait a longer time for them to be functional. High ECM density reduced T cells viability favoring CD4^+^ T cells over CD8^+^ T cells, already after 24 h of culture. This result supports the paradigm that tumor matrices selectively elude the intervention of cytotoxic CD8^+^ cells, while they do not act on CD4^+^ cells, whose task is to maintain the elevated inflammatory state [39].

Another important statement we made was that T cells indeed respond to their extracellular matrix environment, with a quick alteration of gene expression in 3D cultured cells compared to 2D control cultures in suspension. NF-kB has long been recognized as a master regulator of the inflammatory response associated with most incident cancers. NF-kB members act as homo or hetero-dimers of p65 (RelA), c-Rel, RelB, p52 (NFKB2) and p50 (NFKB1) subunits, whose gene transcription is tightly regulated to achieve correct activation of two major pathways, the canonical involving p65/p50, and the non-canonical involving p52 and RelB [40]. The role of NF-kB in the tumor immune response is ambivalent, depending on the type of immune cells infiltrating the tumor, so that its increased expression in CD4^+^ lymphocytes is correlated to the regulatory T cell subtype development and to the inhibition of anti-tumor immune response [41], while anergic CD8^+^ T cells, associated with impaired antitumor immunity, present ablated NF-kB activation [42]. Our gene expression data clearly depict this opposite effect of cancer TME mostly observed in activated cells. In fact, while CD4^+^ lymphocytes upregulate *A20* and *NFKB1* genes over-time when embedded in tumor 3D constructs, CD8^+^ lymphocytes immediately downregulate most NF-kB genes. CD8^+^ also reduced *SP1* expression at 24 h, suggesting that the two transcription factors cooperate to cell transcriptional reprogramming as already suggested by Evaristo et al. [43,44].

Most importantly, the sudden effects on NF-kB pathway correlated to a strong reduction of *IFNγ* and *CD73* in CD8^+^ cells, as NF-kB activation is known to increase the number of tumor-specific IFNγ-producing CD8^+^ T cells and is required for tumor elimination [44]. This surprising observation suggests that the mechanical properties of the ECM significantly alter the cytotoxic activity. This implies that 3D culture models, which more accurately mimic tissue environments, could be highly relevant to recapitulate T-cell physiology and structure studies. Although the 3D culture of T cells, compared to the normal 2D method, has led to a substantial change in the transcriptional profile, to appreciate the changes in membrane markers it is necessary to wait for more cell cycle divisions and maturation times. The effect of ECM density on the cytotoxic capacity of T cells could constitute a novel immunosuppressive mechanism within the tumor microenvironment and provide an explanation for the correlation between the mechanical properties of ECM in tumors and the prognosis of cancer patients. Therefore, the collagen density of the TME may support tumor cell escape from immune destruction by reducing defined T cell viability and modulating the cytotoxic activity of tumor infiltrating immune cells. The identified immunosuppressive mechanism may be relevant during tumor progression, but also for the efficacy of cancer immunotherapy. It has been suggested that the altered migration of T cells to tumor islets is caused by increased immobilization activity prompted by the tumor mass; furthermore, it was speculated that it is due to the secretion of particular factors by the CAF in the TME. These studies further underline that tumor ECM remodeling should be considered an innovative therapeutic target to support the action of the immune system against tumors.

## 4. Materials and Methods

### 4.1. Peripheral Blood Mononuclear Cell (PBMC) Isolation

Buffy coats from three healthy donors, provided by IRCCS Policlinico Ospedale Maggiore (Milan, Italy), were diluted 1:1 with PBS w/o Ca^2+^/Mg^2+^, layered onto Ficoll Paque Plus (Sigma-Aldrich, St. Louis, Missouri, USA) and centrifuged at 2000 rpm for 20 min at room temperature (RT). PBMCs were collected at the interface, washed twice with PBS and centrifuged at 1600 rpm for 10 min at RT. Finally, cells were incubated in BD Pharm Lyse lysing Buffer (1×, BD Biosciences, Franklin Lakes, NJ, USA) for 6 min, washed in PBS and resuspended in RPMI 1640 medium supplemented with 10% FBS, penicillin/streptomycin (100 U/mL/100 μg/mL; Thermo Fisher Scientific, Waltham, Massachusetts, USA ) and 20 U/mL human IL-2 IS (Miltenyi Biotec, Bergisch Gladbach, Germany). T cells were enriched first by magnetic separation (AutoMACS, Miltenyi Biotec, Bergisch Gladbach, Germany) using human Pan T cells isolation kit (Miltenyi Biotec), and then by FACS sorting using a FACS Aria III SORP (BD) according to the expression of specific surface markers. CD4^+^ T cells were isolated by selecting double positive cells for anti-CD3 PB (1:50, Cl.:REA613; Miltenyi Biotec) and anti-CD4 PerCp (1:50, Cl.:L200; BD Biosciences) antibodies, while CD8^+^ T cells choosing the CD3^+^ positive and CD4 negative T cells. Resting T cells were used immediately after purification to carry out the experiments, while T cells Dynabeads human T-Activator CD3/CD28 were used at a bead-to-cell ratio of 1:3 for 48 h to perform the experiment on activated T cells.

### 4.2. Hydrogel Formulation

The PEG-FB choice was dictated by the need to exclude tumor variability due to the different concentrations and proportions of the diverse collagen types, obtaining reliable results on lymphocytes exposed to specific mechanical stimuli.

Poly(ethylene glycol)-fibrinogen (PEG-FB) with a G’ = 385 Pa in a stock concentration of 11 mg/mL was used to generate the 3D bulk constructs. The low stiffness group, simulating the healthy tissue, was obtained formulating a PEG-FB pre-solution at 5 mg/mL concentration in 1× PBS. The cancerous tissue was mimicked using 8 mg/mL PF supplemented with 1% *w*/*v* Polyethylene Glycol Diacrylate (PEG-DA) diluted in 1× PBS. Irgacure 2959 was used as radical photoinitiator at a concentration of 0.01% w/v. Resting and activated CD4^+^ or CD8^+^ lymphocytes were resuspended in each sterile PEG-FB working solutions (5 mg/mL and 8 mg/mL + 1% PEG-DA) at a concentration of 3 × 10^6^ cells/mL, poured in Polydimethylsiloxane (PDMS) mold of 100 μL, and UV cross-linked (365 nm, 4–5 mW/cm^2^) for 5 min. The obtained bulk constructs were divided in the different experimental groups, while lymphocytes, grown in standard suspension cultures, were used as control group. Finally, the samples were processed at early activation time points (0, 12 and 24 h) to perform flow cytometry, gene expression and immunofluorescence assays. The 0 h timepoint referred to bulk samples digested immediately after UV crosslinking.

### 4.3. Biomaterial Pre-Solutions Rheology

Rheological properties of the hydrogel precursor solutions were analysed using a rotational rheometer (Anton Paar RHEOPLUS-32, plate-cone geometry, Turin, Italy). All measurements were performed at 25 °C, and the samples were allowed to reach equilibrium temperature for 10 min prior to each measurement.

### 4.4. Bulk Construct Mechanical Testing—Young’s Modulus

The compressive stress-strain measurements were performed on cylindrical bulk discs of 300 μL of volume (diameter 8mm, height 6mm). Two solutions of PF (5 mg/mL or 8 mg/mL supplemented with 1% of PEG-DA) were cross-linked using a UV light at 365 nm (intensity 4–5 mW/cm^2^) for 5 min. Compression tests were performed with a DMA Q800 Dynamic Mechanical Analyzer (TA Instruments, New Castle, DE, USA) in the strain rate mode. All tests were performed at 25 °C with the following parameters: 0.003 N preloaded force, 5% min—1 strain rate and final deformation was set at 30%. Young’s modulus was calculated from the initial linear regions (0–5% of strain) of obtained stress-strain curves. Each measurement was performed in triplicate and results are reported as the mean ± standard deviation.

### 4.5. Flow Cytometry Analysis

CD4^+^ and CD8^+^ T cells were extracted from the bulks and analyzed by flow cytometry. Briefly, 5 mg/mL PF and 8mg/mL PF + 1% PEG-DA hydrogels were washed in PBS and finely minced using a sterile scissors at the different timepoint. Then, the samples were treated with a digestion solution composed by 300 U/mL Collagenase II (337 U/mg; Worthington) and 0.65 U/mL Collagenase D (0.29 U/mg; Sigma-Aldrich, St. Louis, Missouri, USA) in HBSS buffer for 10 min (5 mg/mL PF bulks) and 25 min (8 mg/mL PF + 1% PEG-DA bulks) at 37 °C. PBS enriched with 5% FBS was used as stop solution to block the digestion process. The cells were centrifuged at 1600 rpm for 5 min at RT. The collected cells were stained for surface marker expression using two different mix of fluorochrome-conjugated monoclonal antibodies: mix 1 was composed of anti-CD3 PB (1:50, cl.REA613; Miltenyi Biotec), anti-CD4-PerCP (1:50, cl.L200; BD Biosciences), anti-CD45RA APC Cy7 (1:50, cl.RPA-T8; Biolegend, San Diego, California, USA), anti-CD27 PE (1:50, cl.M-T271; Biolegend) and anti-CD69 APC (1:50, cl.FN50; Biolegend); mix 2 comprised anti-CD3 PB, anti-CD4 PerCP, anti-PD1 APC Cy7 (1:50, cl.EH122H7; Biolegend), anti-CD25 APC (1:50, cl.BC96; Biolegend) and anti-CD127 PE (1:50, cl.REA 614; Biolegend). The cell viability was evaluated through LIVE/DEAD Fixable Aqua Dead Cell Stain Kit (1:1000, Invitrogen, Carlsbad, California, USA). The cell suspensions were incubated for 30 min at 37 °C, washed with PBS and acquired using a FACS Canto II cytometer (Becton Dickinson, BD). The data analysis was performed with FlowJo V10 software.

### 4.6. Immunofluorescence Assay

A Cytospin 4 Cytocentrifuge (Thermo Fisher scientific) was employed to obtain a CD4^+^ and CD8^+^ T cells on the glass slides for the 2D control group since they grow in suspension. The obtained samples were fixed in 4% Paraformaldehyde (PFA) for 15 min and permeabilized in 0.3% TRITON X-100 (Sigma-Aldrich) for 10 min at RT. Then, the samples were incubated in 5% Bovine Serum Albumin (BSA) blocking solution for 30 min to saturate the nonspecific sites, and incubated with Phalloidin Alexa Fluor647 antibody (1:100, Thermo Fisher Scientific) diluted in 0.5% BSA solution overnight at 4 °C.

CD4^+^ or CD8^+^ T cells in 3D culture conditions were fixed in 4% PFA for 3 h, permeabilized with 0.3% TRITON X-100 (Sigma-Aldrich) diluted in PBS for 50 min, and incubated in 5% BSA blocking solution for 1 h at RT. The constructs were incubated in Phalloidin Alexa Fluor647 (1:50, Thermo Fisher scientific) dilution in 0.5% BSA solution overnight at 4 °C.

Thereafter, 2D and 3D samples were washed in 1× PBS. Nuclei were counterstained with Dapi (1:1000) for 1 h and washed twice with 1× PBS.

The immunofluorescence analysis was performed after the cell encapsulation in h3D and t3D bulks (0 h).

### 4.7. Image Acquisition and Processing

NIKON Eclipse Ti microscope with CRESTOptics X-light V2-VCS spinning disk and Andor DU888 EMCCD camera, with SpectraAura Lumencore 6-led excitation lines were used to acquire qualitative images of h3D and t3D samples. For better Z movement using Z-drive instead of piezo MCL and for stacking of large volumes no perfect focus system (PFS) was used (minimum 530-maximum 1340 μm-thickness).

Leica SP5 laser scanning confocal microscope was used to acquire labelled 2D and 3D samples. For 3D bulks were acquired 1–3 areas at 10× (50 steps Z10um) and 6 areas at 10× + 3× zoom (99 steps Z5um). All datasets, both from spinning disk and from laser-scanning acquisition, were further processed and elaborated via NIS-Elements v.5.30 software (Leica Microsystem, Wetzlar, Germany). Richardson-Lucy deconvolution algorithms were performed with specific parameter for acquisition types, over 20 iterations for low noise background. For qualitative representations, median filtering (kernel 3, iterations 2) was performed. Best focal plans and maximum intensity projections were prepared for qualitative images. Volumetric 3D reconstruction was achieved for better in-depth visualization on 500μm^3^ volumes acquired at LSCM SP5 with 10× + 3× Zoom-in. Digital analysis was conducted using the GA3 module, employing machine-learning object segmentation and classification based on signal intensities, size and morphology. Segmented objects were connected in 3D to get volumetric quantifications.

### 4.8. Gene Expression Analysis

Total RNA was extracted from CD4^+^ and CD8^+^ T cells of each experimental condition. The T cells encapsulated in 3D bulks were collected through digestion process as previously described in the flow cytometer analysis paragraph. All samples were incubated in 1 mL of TRIZOL reagent (15,596,026, Invitrogen, Life Technologies) for 5 min at RT to allow complete dissociation of nucleoprotein complexes. A concentration of 0.2 mL of chloroform per ml of TRIZOL was added. Tubes were vigorously shaken for 15 s, incubated at RT for 3 min and centrifuged at 12,000× *g* for 15 min at 4 °C. The aqueous phase was transferred to a fresh tube and RNA was precipitated with 0.5 mL of isopropyl alcohol. After centrifugation, the RNA pellet was washed with 1 mL of 75% ethanol and centrifuged at 7500× *g* for 5 min at 4 °C. Finally, the RNA pellet was air-dried and dissolved in 10 μL of RNase free water. RNA concentration was determined using a NanoDrop UV–visible spectrophotometer. A 1 μg quantity of total RNA was reverse transcribed to cDNA using the High-Capacity cDNA Reverse Transcription Kit (Applied Biosystem, Foster City, California, USA). The evaluation of gene expression was performed by quantitative real-time polymerase chain reaction (qRT-PCR) using 7900HT Fast Real-time PCR System equipped with SDS software (Applied Biosystems) (Table 1). All reactions were performed in 10 μL reaction volume and in duplicate. Fold changes of each target gene compared to the control group was evaluated.

### 4.9. Statistical Analysis

Statistical analysis was carried out using Prism 8 (GraphPad Software, La Jolla, CA, USA). Data are presented as mean ± standard error of mean (SEM). The statistical significance was assessed by the repeated measure ANOVA with Tukey correction to evaluate the differences between means, while Student’s t-test was used to analyze the nuclear size comparisons. A *p*-value <0.033 (*), 0.002 (**) and 0.001 (***) was considered statistically significant.

## Figures and Tables

**Table 1:** Human primer sequences for qRT-PCR analysis.

Gene Symbol	Sense-Forward Primer	Antisense-Reverse Primer
*TBP*	CGGTTTGCTGCGGTA	TGTGCACACCATTTT
*A20*	GAAAACGAACGGTGACGG	GAGACTCCAGTTGCCAGC
*NFKB1*	GTGGAGCACGACAAC	GGTGTGGTTCCATCG
*NFKB2*	TGAAGCCAGTCATCT	CCTGCTGTCTTGTCC
*REL A*	ATCCCATCTTTGACAATCGTGC	CTGGTCCCGTGAAATACACCTC
*REL B*	CATTGAGCGGAAGATTCAAC	GCAGCTCTGATGTGTTTGTG
*SP1*	TGAAGCGCTTAGGAC	GAGCTACAGAGGCACAAACG
*CD73*	GCATTCCTGAAGATCCAAGC	GCATCACAAATCAGGTTGCC
*IFNγ*	TGGCTTTTCAGCTCTGCATC	CCGCTACATCTGAATGACCTG
*PD1*	GAGGGACAATAGGAG	GCTCCCCATAGTCCA

## Data Availability

The data presented in this study are available in insert article and supplementary material. The row data are available on request from the corresponding author.
